# A LGBM model for predicting alimentary tract hemorrhage after intracerebral hemorrhage surgery: association with malnutrition risk and poor neurological recovery

**DOI:** 10.3389/fmed.2026.1723839

**Published:** 2026-01-23

**Authors:** Guohua Li, Shaojie Li, DongXing Su, Wei Huang, Xuehua Wu, Mingya Cai

**Affiliations:** 1 Jinjiang Municipal Hospital (Shanghai Sixth People's Hospital Fujian), Quanzhou, China; 2 The Second Affiliated Hospital of Fujian Medical University, Quanzhou, China

**Keywords:** alimentary tract hemorrhage, intracerebral hemorrhage, machine learning, postoperative complications, prognosis, risk prediction

## Abstract

**Background:**

Alimentary tract hemorrhage (ATH) after intracerebral hemorrhage (ICH) surgery is a common complication that can increase morbidity and mortality. Prevention of this complication is important for recovery of ICH patients, and early identification of high-risk patients would facilitate targeted prevention. Machine learning (ML) is a data-driven tool that can potentially be used to predict postoperative ATH in ICH surgical patients. However, there are currently no validated ML models for this purpose.

**Methods:**

A retrospective cohort study was performed with 658 ICH surgical patients from a single center. Five predictors were selected with the Boruta algorithm, and a total of 12 ML models were developed. The models were validated on a 70/30 train-test split, and further performance validation was performed with 10-fold cross-validation. The primary endpoint was postoperative ATH, and long-term functional outcome at 180 days was assessed with Modified Rankin Scale (MRS).

**Results:**

The Light Gradient-Boosting Machine (LGBM) model showed the best performance with an AUROC of 0.918 in the test set and an average AUROC of 0.949 on cross-validation. The five confirmed predictors were hemorrhage volume, Glasgow Coma Scale (GCS) score, surgery time, albumin, and glucose. In addition, ATH was significantly associated with lower odds of good functional outcome (MRS 0–2) at 180 days (log-rank *p* = 0.0012).

**Conclusion:**

The present study developed an accurate and easy-to-use ML model for early prediction of ATH in ICH surgical patients. Postoperative ATH was associated with worse long-term neurological recovery, further highlighting the importance of its prevention. The developed model should be externally validated and further used to guide the development of personalized prophylactic ATH strategies.

## Background

Intracerebral hemorrhage (ICH) is a catastrophic subtype of stroke, accounting for 10–20% of all cerebrovascular events but contributing disproportionately to stroke-related mortality and disability. The 30-day mortality remains 30–55%, and only a few survivors regain functional independence, resulting in a heavy socioeconomic burden on families and healthcare systems (1). Its high morbidity and mortality are mainly due to hematoma mass effect and secondary injury cascades such as intracranial hypertension and perihematomal edema, which aggravate neuronal damage and increase fatality rates (2). Despite advances in treatment, preventing and predicting post-ICH complications remain challenging. Among systemic complications, alimentary tract hemorrhage (ATH) is common and severe, with an incidence of 10–30% (3). ATH prolongs hospitalization, raises infection risk, and nearly doubles in-hospital mortality. Mechanisms include stress-related mucosal injury, elevated intracranial pressure, autonomic dysfunction, and systemic inflammation. Patients with ATH after ICH are prone to multiorgan dysfunction, sepsis, and poor neurological outcomes (1). Even mild ATH may worsen prognosis and reduce survival during recovery (2). Increased intracranial pressure and hematoma volume are independent predictors of poor outcomes, while stress-induced gastrointestinal injury reflects severe neurological insult (4). Thus, identifying early predictors of ATH and poor prognosis is vital for optimizing perioperative management and improving ICH outcomes.

Recent years have witnessed growing efforts to apply predictive modeling to evaluate post-ICH complications; however, traditional regression-based models remain limited by linear assumptions, small sample sizes, and insufficient external validation. For instance, Liu et al. constructed a logistic regression-based nomogram incorporating gastric pH, hematoma volume, and sepsis, achieving moderate discrimination but restricted generalizability (5). In contrast, advanced machine-learning (ML) algorithms such as random forest, XGBoost, and CatBoost have shown superior nonlinear fitting capability and improved interpretability in predicting ICH-related complications, including stroke-associated pneumonia and poor functional outcomes (6). Other studies have applied multi-algorithmic frameworks to optimize feature selection and enhance robustness across clinical datasets (7). Furthermore, recent population-based analyses demonstrated that integrating cross-validation and sensitivity testing substantially improves model stability and clinical translation (8). Building upon these advances, our study introduces a comprehensive 12-algorithm ML pipeline with 10-fold cross-validation to identify the optimal model predicting postoperative alimentary tract hemorrhage in ICH patients. By integrating sensitivity analysis, trend testing, and correlation modeling, this approach overcomes the linear and single-center limitations of prior studies and provides novel insights into nonlinear risk thresholds and prognostic implications, thus filling an important gap in current ICH complication-prediction research.

## Methods

### Study population

**Figure 1:**
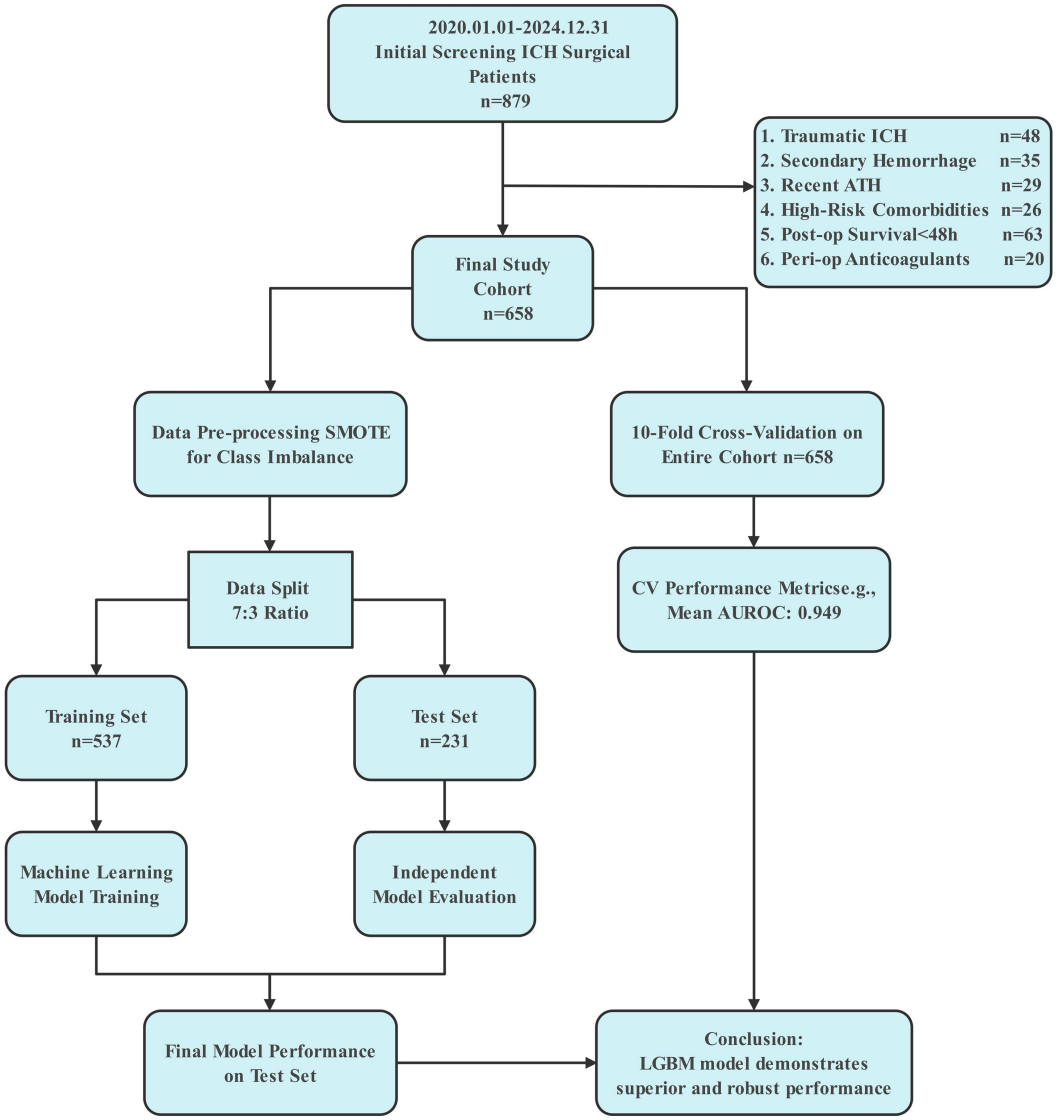
Study flowchart and machine learning pipeline for predicting postoperative alimentary tract hemorrhage. The flowchart illustrates the patient selection process and the analytical methodology. From an initial screening of 879 intracerebral hemorrhage (ICH) surgical patients, 658 were included in the final study cohort after applying exclusion criteria. The machine learning (ML) pipeline involved 10-fold cross-validation on the entire cohort, followed by data pre-processing using SMOTE (synthetic minority over-sampling technique) to address class imbalance. The data was then split into a training set (70%) and an independent test set (30%). Multiple ML models were trained and evaluated, with the light gradient-boosting machine (LGBM) model identified as the best performer.

This single-center, retrospective cohort study initially screened 879 consecutive patients who underwent surgical evacuation for ICH at Jinjiang Hospital between January 1, 2020, and December 31, 2024. The final study cohort comprised 658 patients who met the inclusion criteria: (1) Aged 18 years or older. (2) Primary admission diagnosis of spontaneous, non-traumatic ICH, confirmed by computed tomography (CT). (3) Underwent a neurosurgical procedure for ICH evacuation or decompression, including but not limited to: craniotomy and hematoma evacuation, minimally invasive surgery (e.g., endoscopic evacuation, stereotactic aspiration) and decompressive craniectomy. (4) Availability of complete pre-operative and post-operative clinical data within the electronic health record (EHR) for the variables of interest (e.g., demographics, Glasgow Coma Scale (GCS), medication history, lab values, imaging data). After applying stringent exclusion criteria: (1) Traumatic ICH (*n* = 48); (2) Hemorrhage secondary to structural lesions such as aneurysm or arteriovenous malformation (*n* = 35); (3) History of ATH within 3 months prior to admission (*n* = 29); (4) coagulopathy or end-stage liver disease (*n* = 26); (5) Postoperative survival of less than 48 h (*n* = 63); (6) Use of potent anticoagulants with incomplete reversal prior to surgery (*n* = 20). The detailed patient selection flowchart is shown in Figure 1.

The rationale for these exclusion criteria is to create a more homogeneous cohort of patients with spontaneous ICH and to reduce the effect of other possible confounding risk factors that have their own independent effect on the development of ATH. Traumatic ICH was excluded from the analysis because of its different pathophysiology, management, and prognosis. ICH secondary to structural lesions (such as arteriovenous malformations or aneurysms) was also excluded from the study for the same reason. Recent ATH (within 1 month of ICH) was excluded to avoid any possibility of preoperative ATH being misclassified as postoperative ATH (after surgery, ATH is considered a new-onset complication). Patients with coagulopathy, end-stage liver disease, or incompletely reversed potent anticoagulants were also excluded because they have a higher systemic risk of bleeding that is independent of neurological injury. Patients with postoperative survival of less than 48 h were also excluded to reduce outcome misclassification (they may not survive long enough for postoperative ATH to develop or to be diagnosed).

### Definition of postoperative ATH

Postoperative ATH was defined as a clinically significant event occurring >24 h after ICH surgery, consistent with stress ulcer prophylaxis guidelines (9). Diagnostic criteria included: (1) overt bleeding (hematemesis, melena, or bloody gastric aspirate); (2) hemodynamic instability with unexplained hemoglobin drop (≥20 g/L) requiring intervention; or (3) endoscopic confirmation of active bleeding (Forrest Ia–IIb) (10–12).

### Ethical statement

This retrospective cohort study was conducted in accordance with the Declaration of Helsinki and received approval from the Institutional Review Board of Jinjiang Hospital. The requirement for informed consent was waived by the ethics committee due to the use of anonymized retrospective data, which posed no more than minimal risk to participants. All patient information was de-identified prior to analysis to ensure confidentiality and privacy.

### Surgical procedure

All patients underwent a neurosurgical procedure for ICH evacuation or decompression. The specific surgical technique—including craniotomy with hematoma evacuation, minimally invasive surgery (e.g., endoscopic evacuation or stereotactic aspiration), or decompressive craniectomy—was selected based on the neurosurgeon’s assessment of the patient’s clinical condition and hematoma characteristics, in accordance with contemporary guidelines.

### Data collection

Pre-operative and post-operative clinical data were systematically extracted from the electronic health records (EHR). The collected variables included demographics (age, gender), clinical scores GCS, vital signs, laboratory parameters (electrolytes, albumin, complete blood count), radiological findings (hemorrhage volume), and comorbidities (hypertension, diabetes mellitus) at admission.

### Follow-up

The primary outcome for long-term analysis was functional status at 180 days post-operation, assessed using the Modified Rankin Scale (MRS). A good functional outcome was defined as an MRS score of 0–2, while a score of 3–6 was categorized as a poor outcome. Follow-up data were obtained via structured telephone interviews or clinical outpatient visits.

### Statistical analysis

Continuous variables were presented as mean ± standard deviation and compared using Student’s t-test or Mann–Whitney U test, as appropriate. Categorical variables were expressed as frequencies and percentages and compared using the Chi-square test. Feature selection was conducted with the Boruta algorithm, a wrapper-based feature selection method that uses random forest model as the meta-algorithm. This method was used to select the most relevant and predictive variables on preoperative ATH before the model development to avoid overfitting. Surgery Time, GCS, Hemorrhage Volume, Albumin and Glucose were selected as the most statistically significant predictors by Boruta and used for machine learning model construction. To ensure a fair comparison across algorithms, we used an identical feature set for all models. Twelve ML models, including Light Gradient-Boosting Machine (LGBM), were developed to predict ATH. The dataset was split into a training set (70%) and a test set (30%), with SMOTE (Synthetic Minority Over-sampling Technique) applied to address class imbalance. Model performance was evaluated using accuracy, recall, F1-score, Matthews Correlation Coefficient (MCC), and Area UNDER the Receiver Operating Characteristic Curve (AUROC). The robustness of the optimal model was validated via 10-fold cross-validation. In order to improve model interpretability, SHapley Additive exPlanations (SHAP) values were calculated for the final LGBM model. The SHAP analysis was applied on the training set only to provide a measure of the magnitude and directionality of the contributions of each predictor to the model output, while preventing information leakage from the test set. The association between the model’s predicted risk and the actual outcome was further examined using multiple logistic regression and trend tests. The relationship between ATH and long-term functional outcome was analyzed using Kaplan–Meier curves with a log-rank test. A two-tailed *p*-value < 0.05 was considered statistically significant. All analyses were performed using Python (version 3.13). With pandas and numpy for data preprocessing and management; scikit-learn for model development, cross-validation, and performance evaluation; imbalanced-learn for SMOTE-based resampling; lightgbm, xgboost, and catboost for gradient boosting models; and matplotlib and seaborn for data visualization, including ROC curves and calibration plots.

## Result

### Model development and validation workflow

The model development and validation process, outlined in Figure 1, began with an initial cohort of 879 intracerebral hemorrhage (ICH) surgical patients which were the utilized data from hospitalized patients at Jinjiang Hospital covering the period from January 1, 2020 to December 31, 2024. After the application of stringent exclusion criteria, 658 patients constituted the final study cohort for analysis. This cohort was utilized in a comprehensive machine-learning pipeline, which first employed a 10-fold cross-validation to assess model stability. To address class imbalance, the Synthetic Minority Over-sampling Technique (SMOTE) was applied, and the data was subsequently partitioned into a training set (*n* = 537, 70%) and an independent test set (*n* = 231, 30%). Following model training and independent evaluation, the Light Gradient-Boosting Machine (LGBM) emerged as the superior model, demonstrating robust predictive performance on the hold-out test set.

### Baseline characteristics of the study population

Of the 658 ICH patients studied, 101 (15.3%) developed postoperative ATH. As detailed in Table 1, patients in the ATH group presented with significantly more severe initial neurological injury, evidenced by lower GCS scores (7.91 ± 2.91 vs. 9.66 ± 3.44, *p* < 0.001) and larger hemorrhage volumes (42.23 ± 24.87 mL vs. 31.58 ± 24.27 mL, *p* < 0.001). They also exhibited a pronounced systemic inflammatory state, with higher white blood cell and neutrophil counts (*p* < 0.01), alongside significantly lower serum albumin levels (36.76 ± 6.18 g/L vs. 39.36 ± 5.57 g/L, *p* < 0.001).

**Table 1:** Baseline characteristics of the study population.

Variable	Overall	Non-ATH	ATH	*p*-value
	*N* = 658	*N* = 557	*N* = 101	
Age	57.81 ± 11.31	57.43 ± 11.17	59.91 ± 11.88	0.054
Time to onset	5.21 ± 5.40	5.22 ± 5.57	5.20 ± 4.35	0.969
GCS	9.39 ± 3.42	9.66 ± 3.44	7.91 ± 2.91	<0.001
K	3.51 ± 0.48	3.51 ± 0.48	3.49 ± 0.49	0.672
Na	137.96 ± 7.63	137.82 ± 8.18	138.72 ± 3.21	0.057
Ca	2.32 ± 0.17	2.33 ± 0.17	2.29 ± 0.15	0.039
P	1.08 ± 3.41	1.12 ± 3.71	0.85 ± 0.31	0.09
Mg	0.85 ± 0.13	0.85 ± 0.14	0.85 ± 0.12	0.903
GLU	9.92 ± 36.30	10.12 ± 39.43	8.81 ± 3.12	0.439
Albumin	38.96 ± 5.74	39.36 ± 5.57	36.76 ± 6.18	<0.001
WBC	11.12 ± 4.95	10.83 ± 4.77	12.74 ± 5.61	0.002
Neutrophile	9.01 ± 4.82	8.75 ± 4.60	10.49 ± 5.68	0.004
Lymphocyte	1.47 ± 1.12	1.46 ± 1.09	1.52 ± 1.23	0.623
Monocyte	0.55 ± 0.40	0.54 ± 0.41	0.64 ± 0.34	0.011
Hb	146.31 ± 21.28	146.96 ± 20.40	142.74 ± 25.39	0.117
PLT	202.04 ± 80.91	200.29 ± 80.30	211.65 ± 83.96	0.21
Hemorrhage volume	33.21 ± 24.64	31.58 ± 24.27	42.23 ± 24.87	<0.001
LOS	22.01 ± 14.58	20.83 ± 14.62	28.52 ± 12.50	<0.001
Surgery time	87.97 ± 75.42	81.28 ± 74.29	124.85 ± 71.13	<0.001
Gender				0.313
Male	427.00 (64.89%)	357.00 (64.09%)	70.00 (69.31%)	
Female	231.00 (35.11%)	200.00 (35.91%)	31.00 (30.69%)	
HP				0.003
No	153.00 (23.25%)	141.00 (25.31%)	12.00 (11.88%)	
Yes	505.00 (76.75%)	416.00 (74.69%)	89.00 (88.12%)	
DM				0.398
No	577.00 (87.69%)	491.00 (88.15%)	86.00 (85.15%)	
Yes	81.00 (12.31%)	66.00 (11.85%)	15.00 (14.85%)	
Smoking				<0.001
No	509.00 (77.36%)	450.00 (80.79%)	59.00 (58.42%)	
Yes	149.00 (22.64%)	107.00 (19.21%)	42.00 (41.58%)	
Drinking				0.183
No	574.00 (87.23%)	490.00 (87.97%)	84.00 (83.17%)	
Yes	84.00 (12.77%)	67.00 (12.03%)	17.00 (16.83%)	
MRS group				0.191
No	280.00 (42.55%)	243.00 (43.63%)	37.00 (36.63%)	
Yes	378.00 (57.45%)	314.00 (56.37%)	64.00 (63.37%)	

ATH, Alimentary Tract Hemorrhage; WBC, White Blood Cell; K, Kalium; Na, Sodium; Ca, Calcium; P, Phosphate; Mg, Magnesium; GLU, Glucose; Hb, Hemoglobin; PLT, Platelets; DM, Diabetes Mellitus; HP, Hypertension; LOS, Length of Stay; MRS, Modified Rankin Scale; GCS, Glasgow Coma Scale.

Furthermore, the development of ATH was associated with a more complicated clinical course. These patients required significantly longer surgical times (124.85 ± 71.13 vs. 81.28 ± 74.29 min, *p* < 0.001) and had a prolonged hospital length of stay (LOS) (28.52 ± 12.50 vs. 20.83 ± 14.62 days, p < 0.001). Comorbidities and habits also differed, with hypertension and smoking being significantly more prevalent in the ATH group (*p* = 0.003 and p < 0.001, respectively). These findings identify a distinct high-risk patient phenotype characterized by greater disease severity, systemic inflammation, and specific comorbidities.

### Identification of significant predictors via feature selection

**Figure 2:**
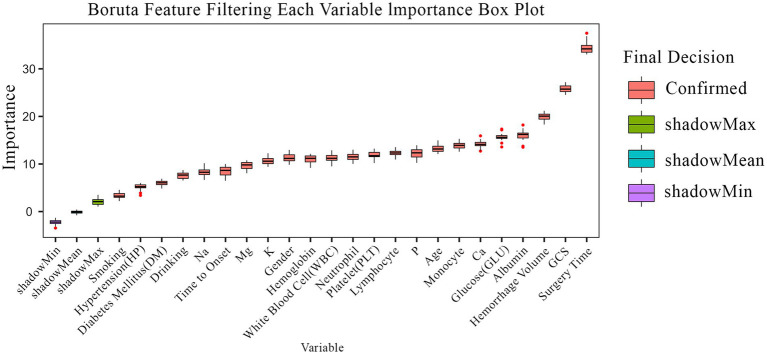
Boruta algorithm for feature selection. This box plot illustrates the importance distribution of all candidate predictors for postoperative alimentary tract hemorrhage, as determined by the Boruta feature selection algorithm. The importance score (*y*-axis) of each clinical variable (*x*-axis) is calculated from multiple iterations of a random forest algorithm. The three horizontal dashed lines represent the maximum, mean, and minimum importance of the shadow features (randomized variables), which serve as a benchmark for significance. A variable is “Confirmed” as important (typically shown in green) if its median importance consistently exceeds the maximum importance of the shadow features (shadowMax). This process identifies a non-redundant set of statistically significant predictors, such as Surgery Time, GCS, Hemorrhage Volume (HV), Albumin, and Glucose (GLU) for inclusion in the final prediction model.

The results of the Boruta feature selection algorithm, detailed in Figure 2, identified a parsimonious set of highly relevant predictors for postoperative ATH. The analysis confirmed that 23 key variables consistently demonstrated importance scores exceeding those of the maximum shadow feature. Based on the feature selection results, the variables confirmed as significant predictors were assessed for multicollinearity. The Variance Inflation Factor (VIF) was calculated for all selected features, and all values were well below the threshold of 5 (range: 1.06–1.69), indicating the absence of significant multicollinearity. The five variables with the smallest VIF values - GCS, Hemorrhage Volume (HV), Albumin, Glucose (GLU) and Surgery Time - were subsequently used to construct the final prediction model, ensuring both clinical relevance and statistical robustness.

### Comparative performance of machine learning models

The predictive performance of 12 distinct machine learning models for postoperative alimentary tract hemorrhage is summarized in Table 2. The Light Gradient-Boosting Machine (LGBM) model demonstrated superior overall performance, achieving the highest accuracy (0.866), Matthews Correlation Coefficient (MCC: 0.711), and Area Under the Receiver Operating Characteristic Curve (AUROC: 0.918). It maintained an excellent balance between sensitivity and specificity, as evidenced by a high F1-score (0.896) and recall (0.931), coupled with a low false negative rate (FNR: 0.069). The Extreme Gradient Boosting (XGB) model showed strong results as a close competitor, while traditional models like Logistic Regression and Naive Bayes exhibited substantially lower performance.

**Table 2:** The result of machine learning model.

Model name	Accuracy	Prevalence	Recall	F1-Score	MCC	AUROC	Precision	Specificity	FNR	FPR
KNNC Test	0.779	0.623	0.896	0.835	0.517	0.846	0.782	0.586	0.104	0.414
GBDT Test	0.797	0.623	0.882	0.844	0.558	0.799	0.809	0.655	0.118	0.345
AdaBoost Test	0.810	0.623	0.882	0.852	0.588	0.786	0.825	0.690	0.118	0.310
LGBM Test	0.866	0.623	0.931	0.896	0.711	0.918	0.865	0.759	0.069	0.241
Logistic Test	0.680	0.623	0.778	0.752	0.303	0.780	0.727	0.517	0.222	0.483
RF Test	0.762	0.623	0.951	0.833	0.485	0.832	0.741	0.448	0.049	0.552
MLP Test	0.797	0.623	0.924	0.850	0.557	0.846	0.787	0.586	0.076	0.414
NB Test	0.658	0.623	0.715	0.723	0.277	0.731	0.730	0.563	0.285	0.437
CatBoost Test	0.797	0.623	0.931	0.851	0.558	0.915	0.784	0.575	0.069	0.425
XGB Test	0.853	0.623	0.917	0.886	0.682	0.923	0.857	0.747	0.083	0.253
SVM Test	0.714	0.623	0.938	0.804	0.365	0.769	0.703	0.345	0.063	0.655
DecisionTree Test	0.766	0.623	0.958	0.836	0.497	0.775	0.742	0.448	0.042	0.552
Mean_scores	0.773	0.623	0.892	0.830	0.508	0.827	0.779	0.577	0.108	0.423

F1-Score, Harmonic Mean of Precision and Recall; MCC, Matthews Correlation Coefficient; AUROC, Area Under the Receiver Operating Characteristic Curve; FNR, False Negative Rate; FPF, False Positive Rate; KNNC Test, k-Nearest Neighbors; GBDT Test, Gradient Boosting Decision Tree; AdaBoost Test, Adaptive Boosting; LGBM Test, Light Gradient-Boosting Machine; Logistic Test, Logistic Regression; RF Test, Random Forest; MLP Test, Multi-Layer Perceptron; NB Test, Naive Bayes; CatBoost Test, Categorical Boosting; XGB Test, Extreme Gradient Boosting; SVM Test, Support Vector Machine; DesicionTree Test, Decision Tree.

**Figure 3:**
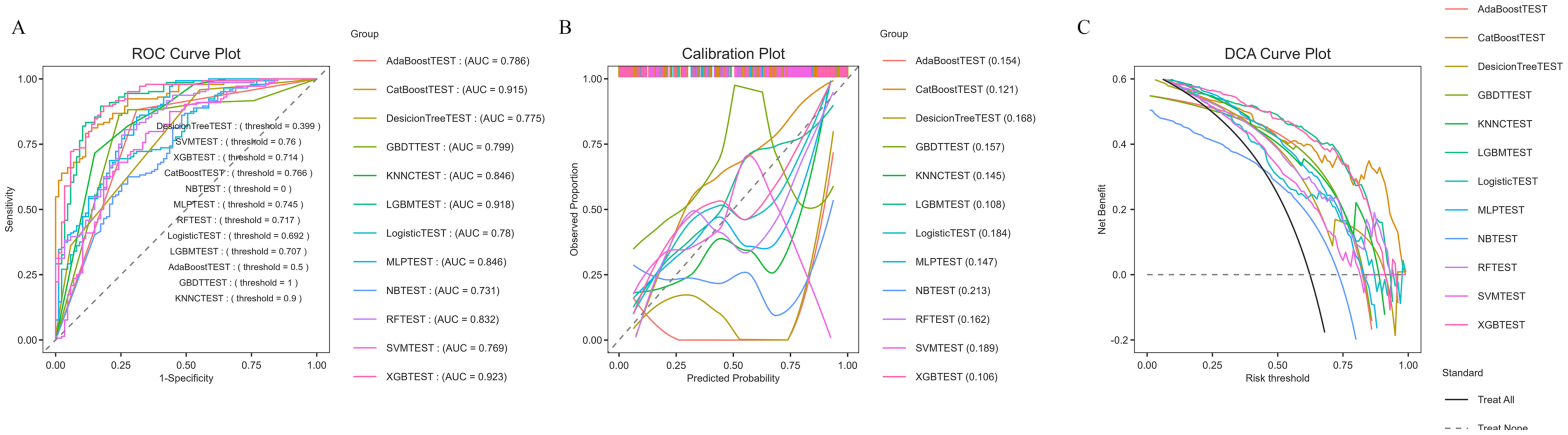
Model discrimination and clinical utility. **(A)** Receiver Operating Characteristic (ROC) curves for the twelve machine learning models predicting postoperative alimentary tract hemorrhage. The corresponding Area Under the Curve (AUC) value for each model is indicated in the legend. The Light Gradient-Boosting Machine (LGBM) model demonstrated the highest discriminatory ability. **(B)** Calibration plots comparing predicted probabilities with observed outcomes in each test cohort. The dashed diagonal line represents perfect calibration. The curves closely follow the reference line, suggesting good agreement between predicted and observed risks. **(C)** Decision Curve Analysis (DCA) evaluating the net clinical benefit of the prediction models. The *y*-axis represents the net benefit, while the *x*-axis represents the probability threshold. The curves for each model are compared against the strategies of intervening in all patients (Treat All) and no patients (Treat None). A model with a higher curve across a range of thresholds offers greater clinical utility.

The comprehensive evaluation of model performance, as depicted in Figure 3, further confirmed the superior predictive power of the LGBM model. The Receiver Operating Characteristic (ROC) curve analysis demonstrated that the LGBM classifier achieved the highest area under the curve (AUC of 0.918), significantly outperforming all other benchmark models. Furthermore, Decision Curve Analysis (DCA) revealed that the LGBM model provided the greatest clinical net benefit across a wide range of probability thresholds, establishing it not only as a statistically powerful tool but also as one with substantial potential for informing clinical decision-making in identifying high-risk patients.

### Model robustness, discriminatory power, and clinical utility

The dataset was randomly partitioned into training set (70%) and an independent test set (30%). All candidate models were then trained using the training set and initially compared on the test set. In addition, to provide a more detailed assessment of model stability and to mitigate the risk of overfitting, a 10-fold cross-validation procedure was later conducted in the entire dataset to assess the robustness and consistency of the final LGBM model. The model demonstrated consistently high performance across all validation folds, with mean scores for accuracy (0.896), recall (0.959), F1-score (0.921), and MCC (0.774) aligning closely with its initial test performance. The mean AUROC of 0.949, with individual folds achieving values up to 0.985, underscored its exceptional and stable discriminatory power. Furthermore, the low false negative rate (0.041) highlighted its reliability in identifying high-risk patients, a critical feature for clinical application (Table 3).

**Table 3:** The result of LGBM test cross-validation.

Model name	Accuracy	Prevalence	Recall	F1-Score	MCC	AUROC	Precision	Specificity	FNR	FPR
LGBM_1Test	0.868	0.658	0.940	0.904	0.702	0.882	0.870	0.731	0.060	0.269
LGBM_1Test	0.868	0.658	0.940	0.904	0.702	0.882	0.870	0.731	0.060	0.269
LGBM_2Test	0.921	0.618	0.979	0.939	0.834	0.972	0.902	0.828	0.021	0.172
LGBM_3Test	0.895	0.605	1.000	0.920	0.790	0.964	0.852	0.733	0.000	0.267
LGBM_4Test	0.934	0.684	0.981	0.953	0.846	0.970	0.927	0.833	0.019	0.167
LGBM_5Test	0.921	0.618	0.957	0.938	0.832	0.979	0.918	0.862	0.043	0.138
LGBM_6Test	0.921	0.579	0.977	0.935	0.840	0.984	0.896	0.844	0.023	0.156
LGBM_7Test	0.816	0.618	0.936	0.863	0.605	0.896	0.800	0.621	0.064	0.379
LGBM_8Test	0.921	0.592	0.933	0.933	0.837	0.963	0.933	0.903	0.067	0.097
LGBM_9Test	0.895	0.658	0.960	0.923	0.763	0.985	0.889	0.769	0.040	0.231
LGBM_10Test	0.895	0.658	0.940	0.922	0.763	0.965	0.904	0.808	0.060	0.192
Mean_scores	0.896	0.632	0.959	0.921	0.774	0.949	0.887	0.787	0.041	0.213

F1-Score, Harmonic Mean of Precision and Recall; MCC, Matthews Correlation Coefficient; AUROC, Area Under the Receiver Operating Characteristic Curve; FNR, False Negative Rate; FPF, False Positive Rate; LGBM Test, Light Gradient-Boosting Machine.

**Figure 4:**
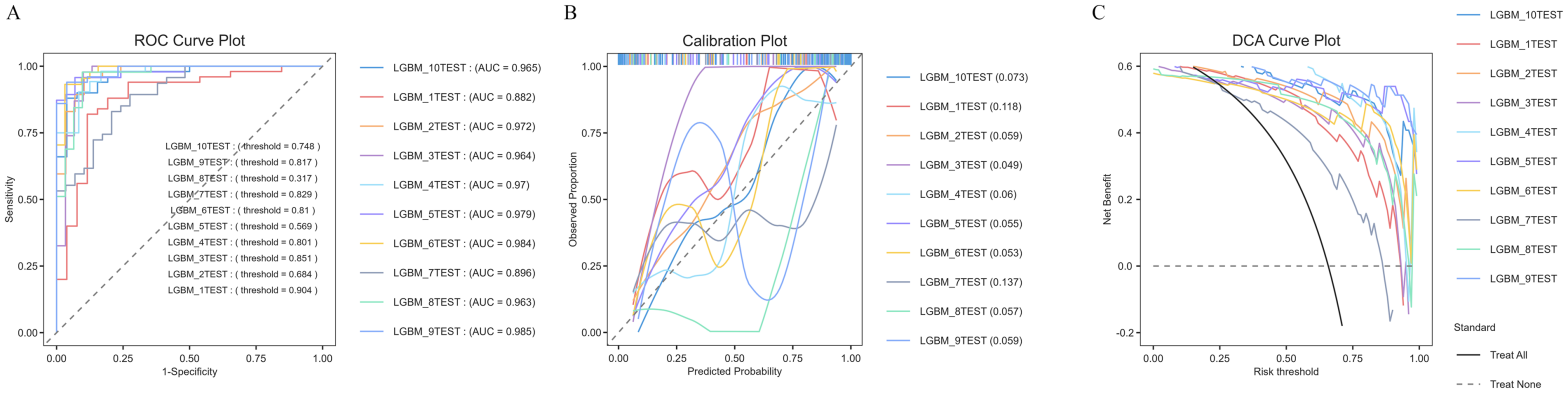
Evaluation of the predictive performance and clinical utility of the LightGBM models in 10 independent test cohorts. **(A)** Receiver operating characteristic (ROC) curves showing the discrimination performance of 10 LightGBM models (LGBM_1TEST–LGBM_10TEST). Each curve corresponds to one test dataset, with area under the curve (AUC) values ranging from 0.882 to 0.985, indicating excellent discrimination across all cohorts. **(B)** Calibration plots comparing predicted probabilities with observed outcomes in each test cohort. The dashed diagonal line represents perfect calibration. The curves closely follow the reference line, suggesting good agreement between predicted and observed risks. **(C)** Decision curve analysis (DCA) plots illustrating the net clinical benefit of the 10 LGBM models across a range of threshold probabilities. All models show greater net benefit than the “treat all” or “treat none” strategies, supporting their potential clinical applicability for individualized risk assessment.

As visually supported by Figure 4, the model exhibited excellent discrimination, calibration, and clinical utility. The ROC curves (Figure 4A) showed consistently high AUC values (0.882–0.985) across test cohorts, while the calibration plots (Figure 4B) revealed strong agreement between predicted probabilities and observed outcomes. The DCA (Figure 4C) further demonstrated that the model provided superior net clinical benefit compared to default “treat-all” or “treat-none” strategies across a wide range of risk thresholds, confirming its potential to inform individualized clinical decision-making.

### Non-linear relationship and risk threshold identification

**Figure 5:**
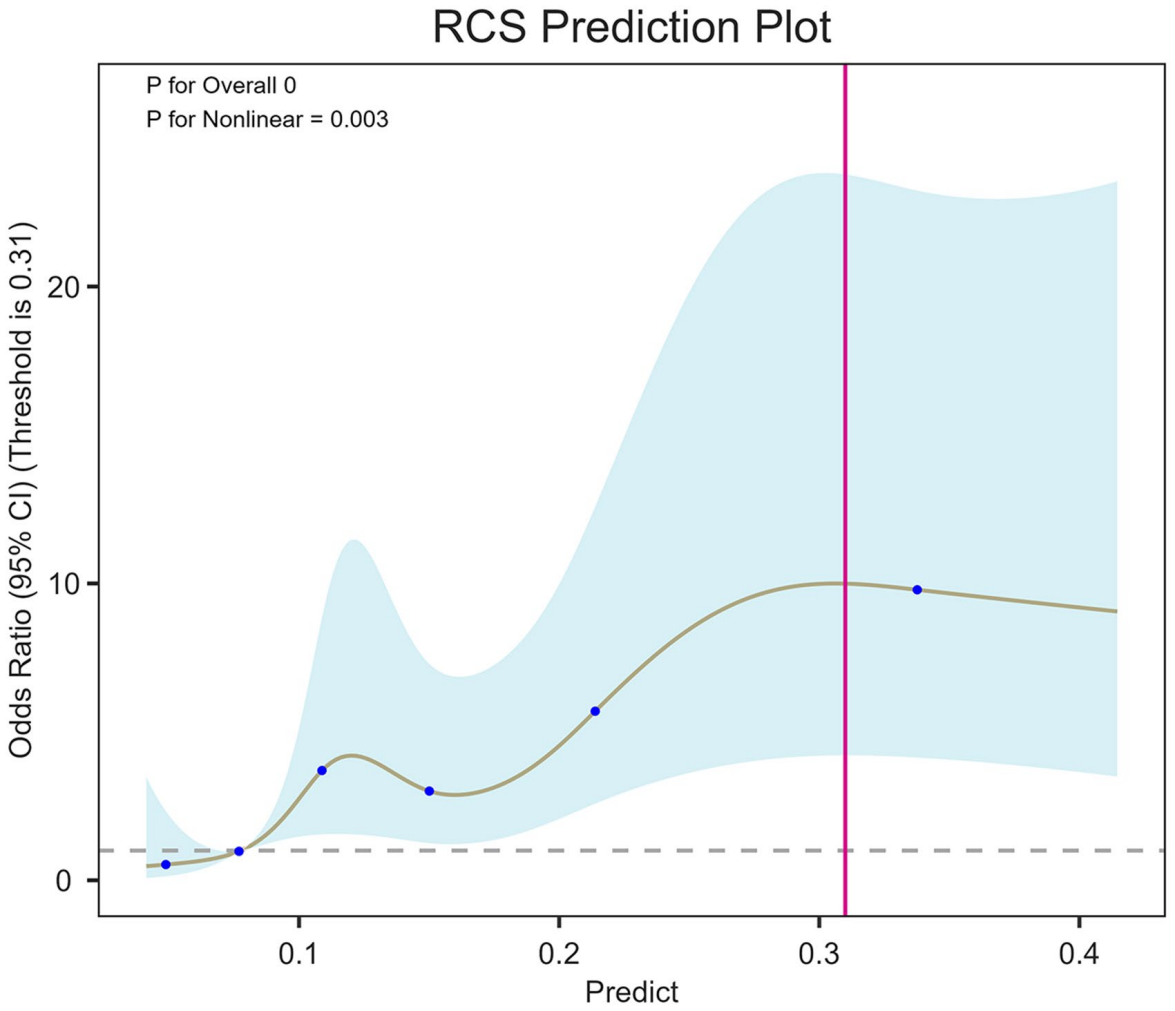
Non-linear association between LGBM model predictor variable and the risk of postoperative alimentary tract hemorrhage. The restricted cubic spline (RCS) plot illustrates the relationship between LGBM model predictor variable and the predicted probability of alimentary tract hemorrhage. The solid curve represents the adjusted odds ratio, with the shaded band indicating the 95% confidence interval. The reference line (dashed) is set at an odds ratio of 1. The overall *p*-value (< 0.001) confirms a significant association, while the non-linear *p*-value (0.003) indicates that the relationship is not linear.

The Restricted Cubic Spline (RCS) analysis, depicted in Figure 5, revealed a significant non-linear relationship between the LGBM model’s predicted risk score and the actual probability of alimentary tract hemorrhage (Overall *p* < 0.001, Non-linear *p* = 0.003). The curve demonstrated a sharp, non-linear increase in hemorrhage risk beyond a specific model prediction score threshold, indicating that the risk does not rise in a simple linear fashion but accelerates markedly after a critical point. This finding allowed for the identification of a clear risk threshold (0.31), which is crucial for stratifying patients into distinct risk categories and defining actionable intervention points in a clinical setting.

### Model interpretation using SHAP values

**Figure 6:**
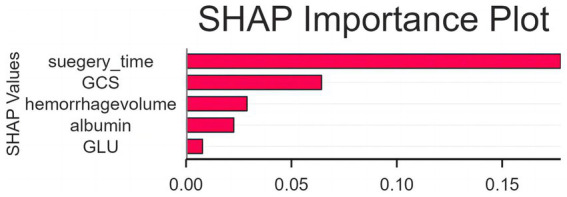
SHAP-based interpretation of the LGBM model. Shapley additive explanations (SHAP) was used to provide additional explainability for the final LGBM model. The bar plot shows the mean absolute SHAP values for each predictor, representing the relative magnitude of each predictor’s contribution to the predicted risk for postoperative alimentary tract hemorrhage. The SHAP value is a normalized value, and the higher the SHAP value, the larger the contribution to the model output. Surgery time has the largest contribution, followed by Glasgow Coma Scale (GCS) and hemorrhage volume, while albumin and glucose exert smaller but consistent effects. These results present a transparent, clinically intuitive approach to describe how perioperative neurological severity, surgical stress, and nutritional–metabolic status contribute to model prediction.

The SHAP importance plot describes the impact of each predictor on the output of the LGBM model (Figure 6). The larger the SHAP value, the greater the contribution of the feature to the model output, with positive values indicating an increase in the predicted risk of postoperative alimentary tract hemorrhage. As in the plot, surgery time had the largest overall impact on risk estimation, followed by neurological injury severity (as measured by GCS and hemorrhage volume). Albumin and glucose had smaller, but more consistent contributions to model output that likely reflect nutritional–metabolic reserve and a stress response to injury, respectively. These findings offer an intuitive interpretation for the relative contributions of perioperative neurological injury, surgical stress, and metabolic status to the model’s estimation of hemorrhage risk.

### Association between LGBM model prediction value and clinical outcome

As detailed in Table 4, both multiple regression and trend analyses confirmed a strong, dose–response relationship between the LGBM model’s predicted value and the risk of ATH. When treated as a continuous variable, the predicted value was an extreme, statistically significant predictor of hemorrhage risk across non-adjusted and adjusted models [Adjust I 648.675 (83.514, 5038.411), *p* < 0.001, Adjust II OR: 121.23, 95% CI: 4.98–2951.13, *p* = 0.003]. When patients were stratified by predicted value quartiles (Q1-Q4), a significant increasing trend was observed (P for trend < 0.001). Specifically, patients in the highest risk quartile (Q4) had markedly elevated odds of hemorrhage compared to the lowest quartile (Q1), with an adjusted odds ratio of 11.53 (95% CI: 3.36–39.58) in the fully adjusted model. This demonstrates that the model’s output is a robust and independent predictor, effectively stratifying patients into distinct risk categories.

**Table 4:** LGBM test predicted value multiple regression and trend test.

Exposure	Non-adjusted		Adjust I		Adjust II	
	OR (95%CI)	*p*-value	OR (95%CI)	*p*-value	OR (95%CI)	*p*-value
Predicted value	368.474 (50.872, 2668.920)	<0.001	648.675 (83.514, 5038.411)	<0.001	121.229 (4.980, 2951.126)	0.00322
Predicted value (IQR)						
Q1	1		1		1	
Q2	4.106 (1.619, 10.412)	0.00293	4.069 (1.597, 10.367)	0.00327	4.111 (1.544, 10.947)	0.00467
Q3	4.106 (1.619, 10.412)	0.00293	4.248 (1.665, 10.837)	0.00247	3.451 (1.153, 10.323)	0.02673
Q4	11.855 (4.920, 28.566)	<0.001	13.234 (5.440, 32.192)	<0.001	11.534 (3.362, 39.576)	<0.001
P for Predicted group trend	11250.121 (664.886, 190356.413)	<0.001	21467.137 (1175.160, 392149.174)	<0.001	10593.147 (89.972, 1247218.387)	<0.001

Adjust I model adjust for: Gender; Age.

Adjust II model adjust for: Gender; Age; Hemorrhage Volume; Time to Onset; Hypertension (HP); Surgery Time; Diabetes Mellitus (DM); Smoking; Drinking.

OR, Odds Ratio; CI, Confidence Interval; IOR, Interquartile Range.

### Long-term functional outcomes stratified by complication status

**Figure 7:**
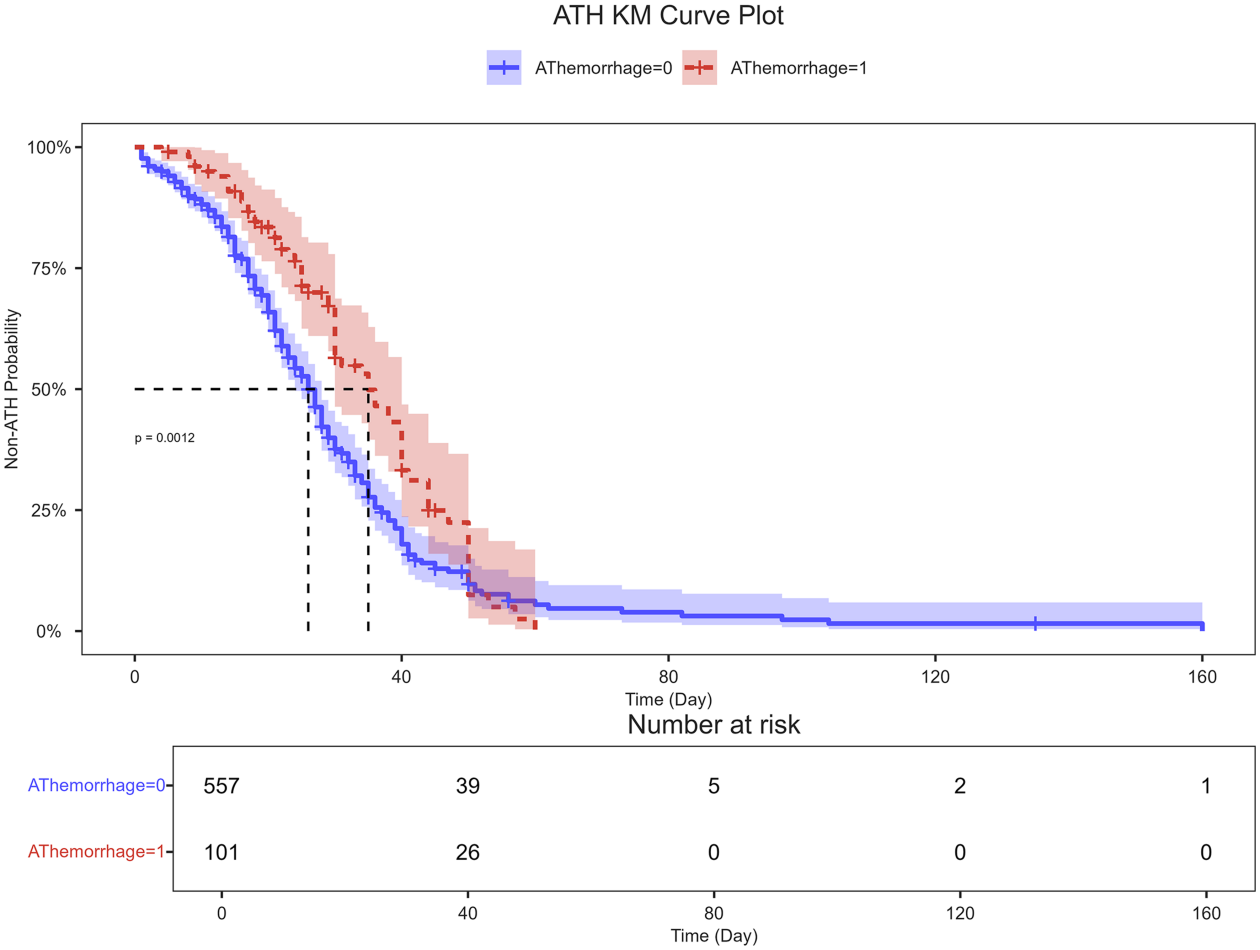
Kaplan–Meier analysis of 180-day functional outcome by ATH status. The Kaplan–Meier curve compares the probability of a good functional outcome (defined as a MRS of 0–2) over 180 days of follow-up between patients who developed postoperative alimentary tract hemorrhage (ATH) and those who did not. The log-rank test was used to compare the two survival curves, with a *p*-value of 0.0012 indicating a statistically significant association between the occurrence of ATH and a lower probability of a good functional outcome. The number of patients at risk at each time point is shown in the table below the plot.

The Kaplan–Meier analysis, presented in Figure 7, demonstrated a significant association between the occurrence of postoperative ATH and poorer long-term functional recovery over the 180-day follow-up period. Patients who developed ATH exhibited a substantially and statistically significant lower probability of achieving a good functional outcome (MRS score 0–2) compared to those without this complication (log-rank *p* = 0.0012). The curves separated early and maintained a consistent divergence, indicating that the negative impact of ATH on neurological recovery was both profound and sustained throughout the convalescent period.

## Discussion

Compared with previous studies, our research provides a more comprehensive and data-driven framework for predicting ATH following ICH. Earlier investigations, such as Misra et al. (13) and Wang et al. (12), mainly focused on limited clinical parameters, such as hematoma size, septicemia, and inflammatory markers, which, while informative, failed to capture complex nonlinear interactions among systemic and neurological variables. Similarly, later works such as Wang et al. (14) and Guo et al. (6) introducted data-driven approaches but were often constrained by small sample sizes, single-center designs, or limited algorithmic diversity. In contrast, our study leveraged 12 machine-learning algorithms with 10-fold cross-validation to ensure model robustness and generalizability, complemented by sensitivity analyses and trend tests that revealed nonlinear relationships and threshold effects between predicted probabilities and observed outcomes. In addition, SHAP-based interpretation enhanced the transparency of the LGBM model by providing an intuitive explanation of how perioperative neurological severity, surgical stress, and nutritional–metabolic status contributed to individual risk predictions, thereby supporting clinician trust in the proposed risk stratification framework. By integrating these methodologies and validating associations with long-term functional outcomes (180-day MRS), our study bridges the gap between traditional statistical inference and modern ML interpretation, thus advancing precision risk stratification for postoperative gastrointestinal bleeding in ICH patients beyond prior frameworks.

The feature importance identified in the ML predictive model—encompassing HV, GCS, Surgery Time, Albumin, and GLU—collectively delineates a coherent pathophysiological narrative through the brain-gut axis framework, integrating neurological injury, systemic stress, and nutritional-metabolic dimensions (15). A substantial HV and consequent low GCS score initiate this cascade via central autonomic network disruption, triggering unchecked vagal stimulation that increases gastric acid secretion and sympathetic overdrive that induces splanchnic vasoconstriction (16, 17). This “brain-initiated attack” on the gut is compounded by prolonged surgery time, which acts as a “second hit,” amplifying neuroendocrine stress and systemic inflammation, thereby extending the duration of mucosal vulnerability (18, 19). Concurrently, hypoalbuminemia reflects not only a state of impaired nutritional reserve but also a marker of systemic inflammation, which diminishes mucosal repair capacity, antioxidant defense, and endothelial integrity (20, 21). Furthermore, hyperglycemia exacerbates this environment by promoting endothelial dysfunction, immune dysregulation, and oxidative stress, creating a milieu that accelerates mucosal injury and impairs healing (22, 23). Crucially, these pathways are bidirectional; the gut responds to this cerebral-insult-induced damage by releasing inflammatory mediators and undergoing microbial composition shifts, which may further propagate neuroinflammation and worsen outcomes, creating a vicious cycle. Thus, the identified predictors embody the core pillars of ATH pathogenesis: severe brain injury (HV and GCS), cumulative stress burden (Surgery Time), and compromised nutritional-metabolic reserve (Albumin, GLU). This integrated model underscores that ATH is not merely a local gastrointestinal event but a manifestation of dysregulated gut-brain crosstalk, highlighting the potential for multimodal interventions that address neurological stability, operative stress minimization, and proactive nutritional-metabolic support to mitigate this serious complication.

The robust association between ATH and poor long-term functional outcomes (MRS) can be fundamentally understood through the gut-brain axis framework, where ATH creates a vicious cycle of bidirectional dysregulation (24, 25). As both a consequence and amplifier of severe brain injury, ATH induces gut-derived inflammation through bacterial translocation and damage-associated molecular patterns, which propagate neuroinflammation via vagal afferents and systemic circulation. Concurrently, ATH critically exacerbates nutritional deficits through blood loss, protein-losing enteropathy, and impaired nutrient absorption, depriving the injured brain of essential substrates for repair—particularly proteins for neurotransmission, iron for oxygen carriage, and micronutrients for antioxidant defense. This dual insult of heightened neuroinflammation and cerebral nutrient deprivation establishes a pathological environment where the brain’s capacity for plasticity and recovery is profoundly compromised, ultimately manifesting as worse functional outcomes. These findings position ATH not merely as a complication but as a pivotal modifier of the brain’s nutritional and inflammatory milieu, highlighting the necessity of integrated nutritional support and gut protection strategies in neurocritical care.

### Advantage and limitation

There are several strengths to our study. We developed a high-performing prediction model using a rigorously curated clinical cohort and well-established machine learning methods. The LGBM model had good discrimination and calibration, as confirmed by internal validation using train–test split and 10-fold cross-validation. The model uses predictors that are easily available and biologically plausible, and the inclusion of long-term functional outcomes demonstrates the clinical importance of postoperative ATH.

There are several limitations to this study that merit discussion. First, the retrospective single-center study design may be subject to selection bias and precludes immediate external validity of our results. Second, we were unable to include several potentially important perioperative and intensive care management variables, such as timing and dosing of stress-ulcer prophylaxis and enteral nutrition, vasopressor use, and ICU treatment strategies, due to lack of consistent availability in structured form in the electronic medical record system. This may lead to residual confounding from unobserved predictors and reflects center-specific practice patterns that may influence model predictions and should be considered during clinical interpretation. Third, algorithm-specific feature expansion and feature engineering was not performed in the single-center derivation cohort. Future studies using multicenter cohorts may consider whether custom feature sets improve model performance for particular architectures. Although the proposed model achieved good discrimination and calibration in internal validation, external validation in independent cohorts is necessary prior to routine clinical implementation. Calibration drift may occur when applying the model to other settings where case-mix, baseline ATH incidence, and perioperative practice patterns may differ. In such cases, model updating approaches such as recalibration of the intercept and slope with local data can be used to better align the predicted risks with observed outcomes while maintaining discrimination. Finally, recent work has shown promising performance of tabular foundation models for structured clinical data, and future large-scale multicenter studies will determine whether these and other approaches can improve upon traditional ML methods (26).

## Conclusion

This study successfully developed and validated a robust LGBM-based ML model that accurately predicts postoperative ATH in ICH patients, demonstrating superior performance through rigorous 10-fold cross-validation. The model’s strengths lie in its use of readily available clinical predictors and its ability to identify high-risk patients, offering significant potential for proactive clinical intervention. Furthermore, the established link between ATH and poorer long-term functional outcomes (MRS) underscores the critical importance of this complication. Future research should focus on external validation in multi-center settings and the development of targeted, model-guided preventive strategies to ultimately improve patient prognosis.

### Clinical implementation

To better illustrate the possible clinical application of the model, we estimate the possible use of the internally derived threshold (0.31) for the risk score in perioperative clinical decision-making. We do not suggest the risk score alone as a trigger for treatment, but rather for providing an additional, pragmatic, and subjective reference that may facilitate the identification of patients for whom closer monitoring and supportive care is indicated.

For patients with a predicted risk score above the threshold, the model could prompt additional clinical scrutiny in the early identification and prevention of postoperative alimentary tract hemorrhage, including closer follow-up of hemoglobin trends and gastrointestinal symptoms, reassessment of stress-ulcer prophylaxis, and early review of nutritional and metabolic status. When indicated, involvement of other clinical specialties, such as intensive care or gastroenterology, may be considered to guide individualized plans for prevention.

For patients predicted as lower risk according to the threshold, current standard perioperative and intensive care pathways can proceed, including usual monitoring and reassessment based on evolving clinical conditions. In this way, the proposed threshold may help guide optimization of resource allocation, minimize missed high-risk patients, and support early preventive interventions, while still allowing for clinical discretion. Ultimately, the clinical utility of the threshold requires further validation in prospective and multicenter studies before routine implementation is considered.

## Data Availability

The raw data supporting the conclusions of this article will be made available by the authors, without undue reservation.
